# ER stress arm XBP1s plays a pivotal role in proteasome inhibition-induced bone formation

**DOI:** 10.1186/s13287-020-02037-3

**Published:** 2020-11-30

**Authors:** Dan Zhang, Kim De Veirman, Rong Fan, Qiang Jian, Yuchen Zhang, Li Lei, Holly Evans, Yanmeng Wang, Lei Lei, Baiyan Wang, Ramone A. Williamson, Andrew Chantry, Pengcheng He, Ang Li, Hendrik De Raeve, Karin Vanderkerken, Aili He, Jinsong Hu

**Affiliations:** 1 Department of Cell Biology and Genetics, Xi’an Jiaotong University Health Science Center, No.76 Yanta West Road, Xi’an, 710061 China; 2 Department of Hematology and Immunology, Myeloma Center Brussels, Vrije Universiteit Brussel, 1090 Brussels, Belgium; 3 Department of Oncology, The First Affiliated Hospital, Xi’an Jiaotong University, Xi’an, 710061 China; 4 Sheffield Myeloma Research Team, Department of Oncology and Metabolism, Medical School, University of Sheffield, Sheffield, UK; 5 Department of Clinical Hematology, The Second Affiliated Hospital, Xi’an Jiaotong University, Xi’an, 710004 China; 6 Department of Clinical Hematology, The First Affiliated Hospital, Xi’an Jiaotong University, Xi’an, 710061 China; 7 Key Laboratory of Shaanxi Province for Craniofacial Precision Medicine Research, College of Stomatology, Xi’an Jiaotong University, Xi’an, 710004 China; 8 Department of Pathology, UZ Brussel, Vrije Universiteit Brussel, 1090 Brussels, Belgium

**Keywords:** Proteasome inhibitor, Multiple myeloma, Mesenchymal stem cell, Xbp1s, Osteogenic differentiation

## Abstract

**Background:**

Bone destruction is a hallmark of multiple myeloma (MM). It has been reported that proteasome inhibitors (PIs) can reduce bone resorption and increase bone formation in MM patients, but the underlying mechanisms remain unclear.

**Methods:**

Mesenchymal stem cells (MSCs) were treated with various doses of PIs, and the effects of bortezomib or carfilzomib on endoplasmic reticulum (ER) stress signaling pathways were analyzed by western blotting and real-time PCR. Alizarin red S (ARS) and alkaline phosphatase (ALP) staining were used to determine the osteogenic differentiation in vitro. Specific inhibitors targeting different ER stress signaling and a Tet-on inducible overexpressing system were used to validate the roles of key ER stress components in regulating osteogenic differentiation of MSCs. Chromatin immunoprecipitation (ChIP) assay was used to evaluate transcription factor-promoter interaction. MicroCT was applied to measure the microarchitecture of bone in model mice in vivo.

**Results:**

We found that both PERK-ATF4 and IRE1α-XBP1s ER stress branches are activated during PI-induced osteogenic differentiation. Inhibition of ATF4 or XBP1s signaling can significantly impair PI-induced osteogenic differentiation. Furthermore, we demonstrated that XBP1s can transcriptionally upregulate ATF4 expression and overexpressing XBP1s can induce the expression of ATF4 and other osteogenic differentiation-related genes and therefore drive osteoblast differentiation. MicroCT analysis further demonstrated that inhibition of XBP1s can strikingly abolish bortezomib-induced bone formation in mouse.

**Conclusions:**

These results demonstrated that XBP1s is a master regulator of PI-induced osteoblast differentiation. Activation of IRE1α-XBP1s ER stress signaling can promote osteogenesis, thus providing a novel strategy for the treatment of myeloma bone disease.

## Introduction

Multiple myeloma (MM) is a hematological malignancy characterized by the accumulation of clonal plasma cells in the bone marrow and excessive monoclonal immunoglobulin in the serum and urine [1]. Osteolytic bone disease is one of the most debilitating manifestations of MM. Myeloma bone disease (MBD) is the result of increased destruction of bone and is present in approximately 80% of newly diagnosed MM patients. Patients with MBD develop skeletal complications, including diffuse osteopenia, bone pain, pathological fractures, spinal cord compression, and hypercalcemia, which are significant causes of morbidity and mortality, thus severely affecting the quality of patients’ life and survival [2]. The development of MBD is associated with unbalanced bone remodeling, in which suppressed osteoblast activity and increased osteoclast activity result in promoted bone absorption and impaired new formation [3].

The treatment of MM has changed dramatically in the last decade. The introduction of new therapeutic drugs, including proteasome inhibitors (PIs), immunomodulatory agents, histone deacetylase inhibitors, and monoclonal antibodies, have significantly improved the overall survival of MM patients [2, 4]. Among these new drugs, PIs are the most important classes of agents, and three kinds of PIs, bortezomib, carfilzomib, and ixazomib, have been approved for the treatment of MM. Bortezomib is the first-in-class PI, which chemically binds reversibly to the β5 subunit of the proteasome (PSMB5). In contrast to bortezomib, carfilzomib is second-in-class PI, which selectively and irreversibly binds and inhibits PSMB5. Ixazomib is the first oral PI still selectively and reversibly targeting PSMB5. Notably, targeting proteasome has been implicated in inhibiting bone resorption and promoting bone formation [5–11]. However, to date, it is still unclear as to how proteasome inhibitors directly regulate bone formation.

The endoplasmic reticulum (ER) is an organelle that serves many general functions, including protein synthesis and transport, protein folding, lipid and steroid synthesis, and carbohydrate metabolism. Perturbations of ER function may lead to ER stress, which is a central hub of the cross-compartmental signaling network designed to restore intracellular homeostasis [12]. The ER stress is distinguished by the action of three main pathways, namely IRE1α (inositol-requiring protein 1α)-XBP1 (X box binding protein 1), PERK (PKR-like ER kinase)-ATF4 (activating transcription factor 4), and ATF6 (activating transcription factor 6). Upon activation, downstream XBP1, ATF4, and ATF6α transcription factors are translocated to the nucleus where they transcriptionally regulate the expression of target genes [12]. In this regard, recent studies in MM cells revealed that PI-induced anti-tumor effect is tightly associated with the activation of ER stress, which triggers the expression of fatal pro-apoptotic factors [13].

In this study, we aimed to delineate the mechanisms of PI-induced bone formation. We hypothesize that proteasome inhibition-activated ER stress signaling is tightly involved in the osteoblast differentiation. To test this hypothesis, we first compared the effects of bortezomib and carfilzomib on osteogenesis of normal mouse bone marrow mesenchymal stem cells (mMSCs), MM patient-derived mesenchymal stem cells (MM-MSCs), and an osteoblast precursor cell line MC3T3-E1. Focusing on the changes and the roles of ER stress signaling pathways, we further demonstrated that the activation of the IRE1α-XBP1 ER stress branch is a master regulator of PI-induced osteogenic differentiation.

## Materials and methods

### Drugs

Bortezomib and carfilzomib were bought from LC Laboratories (Woburn, MA, USA). For in vitro studies, the drugs were reconstituted in dimethylsulfoxide at a stock concentration of 10 mM. PERK inhibitor GSK2606414 was bought from MedChemExpress (Monmouth Junction, NJ, USA), and IRE1α inhibitor MKC3946 was bought from BOC Sciences. MKC3946 was diluted in Cremophor EL (Sigma-Aldrich, Bornem, Belgium) and PBS to the appropriate concentration.

### Cells and cell culture conditions

Mouse bone marrow mesenchymal stem cells were isolated from 4 to 6 weeks aged C57BL/6 mice. The mice were housed and treated following conditions approved by the Ethical Committee for Animal Experiments of Xi’an Jiaotong University Health Science Center (No. 2015-123). Details of isolation and expansion of mMSCs are specified in Supplemental Method 1. MSCs derived from MM patients were obtained from the BM samples, cultured and identified following a protocol previously described [14]. MC3T3-E1 cell line was obtained from the Kunming Cell Bank of Type Culture Collection of the Chinese Academy of Sciences. MSCs and MC3T3-E1 cells were cultured in Dulbecco’s modified Eagle’s medium (DMEM) supplemented with 10% fetal bovine serum (FBS) (Biological Industries, Kibbutz Beit-Haemek, Israel) and 100 U/mL penicillin-streptomycin and 2 mM L-glutamine. Cells cultured in an osteogenic induction media containing 50 mg/mL ascorbic acid, 100 nM dexamethasone, and 10 mM β-glycerophosphate sodium were used as positive controls.

### Alizarin red S staining

Alizarin red S (ARS) staining was used to examine the formation of mineralized bone nodules in the various MSC cultures, following a protocol previously described [11]. Images of ARS were captured by using a digital camera (Nikon D200, Tokyo, Japan) or under a phase-contrast microscope (Nikon Eclipse TS100, Tokyo, Japan).

### Alkaline phosphatase activity staining in vitro

The Gomori method was used to stain alkaline phosphatase (ALP), following the manufacturer’s instructions (Heart Biotech, Shaanxi, China). Briefly, cells were fixed with cold isopropanol for 10 min, followed by washing with ultrapure water. The cells were then stained with fresh ALP incubation buffer (2% barbitone sodium, 2% anhydrous calcium chloride, 3% β-sodium glycerol-phosphate, 2% magnesium sulfate, pH 9.4) for 4 h at 37 °C. The cells were rinsed with ultrapure water for 10 min, treated with 2% cobalt nitrate for 5 min, followed by 1% ammonium sulfide solution for 2 min. After drying, the stained cells were imaged by using a digital camera.

### Western blotting

The cells were harvested and lysed with radioimmunoprecipitation assay (RIPA) buffer containing protease inhibitor cocktail and phosphatase inhibitor cocktails (Sigma-Aldrich, Shanghai, China). Cell lysates were then subjected to sodium dodecyl sulfate-polyacrylamide gel electrophoresis (SDS-PAGE), transferred to polyvinylidene difluoride (PVDF) membranes, immunoblotted with various antibodies, and analyzed as described previously [15]. The antibodies and dilutions used for Western blotting are listed in Supplemental Table 1.

### RNA extraction, reverse transcription, and quantitative real-time PCR

RNA extraction, reverse transcription PCR, and real-time PCR were performed as described previously [15]. The relative expression levels of each gene were analyzed using the 2^−ΔΔCt^ method. The sequences of forward and reverse primers for these genes are listed in Supplemental Table 2.

### RT-PCR for Xbp1 splicing assay

A standard RT-PCR analysis was performed to detect spliced *Xbp1* for mouse using the following primers: 5′-ACACGCTTGGGAATGGACAC-3′ (forward) and 5′-CCATGGGAAGATGTTCTGGG-3′ (reverse); for human: 5′-TTGCTGAAGAGGAGGCGGAAG-3′ (forward) and 5′-GGTCCAAGTTGTCCAGAATGC-3′ (reverse). The program was used as follows: (i) 94 °C for 3 min; (ii) 30 cycles of 94 °C for 30 s, 58 °C for 30 s, and 72 °C for 30 s; and (iii) 72 °C for 10 min. The PCR products were separated by 3.5% agarose gel electrophoresis to resolve the 152 bp (unspliced) and 126 bp (spliced) amplicons for mouse *Xbp1* and 184 bp (unspliced) and 210 bp (spliced) for human *XBP1*.

### Chromatin immunoprecipitation (ChIP) assay

A ChIP assay was performed according to the Simple ChIP Enzymatic Chromatin IP Kit protocol (Cell Signaling Technology, Shanghai, China) [15]. Briefly, mMSCs were cultured in the presence of 2.5 nM bortezomib for 1 day. After incubation, cells (1 × 10^7^) were subjected to the ChIP assay. The chromatin was immunoprecipitated with anti-XBP1 (Santa Cruz Biotechnology, Dallas, TX, USA) at a 1:50 dilution and normal mouse IgG (Santa Cruz Biotechnology, USA) at a 1:100 dilution at 4 °C on a rotator for 16 h. The fraction of the purified ChIP DNA or input was used as templates for PCR analysis. PCR primer pair was generated to detect DNA segments located at the promoter of mouse *Atf4*. The nucleotide sequences of the primers used in the assay are presented in Supplemental Table 3.

### Lentiviral particle transduction

Full-length human *XBP1s* cDNA was amplified and cloned into the Tet-On inducible lentiviral vector GV437 (TetIIP-MCS-EGFP-3FLAG-Ubi-TetR-IRES-Puromycin) (Genechem, Shanghai, China), and the construct sequence was verified by sequencing. Lentiviral particles were produced by standard transient transfection of a three-plasmid system into producer cells 293T. For lentiviral infection, mMSCs or MC3T3-E1 cells were seeded overnight; then, the viral particles were added to the plated cells at a multiplicity of infection of 100 in a minimal volume of medium supplemented with 5 μg/mL polybrene for 24 h in order to achieve high transduction efficiency (> 80%), which can be evaluated by monitoring GFP expression in living cells. 2.5 μg/mL of doxycycline was used to induce XBP1s expression for the time indicated. Empty lentiviral vector was used as control.

### Mice and treatment schedule

Five-week old C57BL/KaLwRij mice (Envigo, Horst, the Netherlands) were housed and maintained following the conditions approved by the Ethical Committee for Animal Experiments, Vrije Universiteit Brussel (license No. LA1230281, 18-281-3). Naive C57BL/KaLwRij mice were treated with MKC3946 (100 mg/kg, intraperitoneally, daily) and/or a low dose of bortezomib (0.3 mg/kg, subcutaneously, daily). After 7 days of treatment, mice were sacrificed, and one femur per mouse was defleshed and stored at 70% EtOH for micro-computed tomography (microCT) analysis. A second femur was fixed in zinc fixative for 48 h, decalcified for 48 h, and paraffin-embedded.

### MicroCT analysis

The fixed femurs isolated from the treated and untreated mice were scanned using a SkyScan 1272 microCT (Bruker, Belgium). MicroCT analysis was performed as previously described [16].

### Immunohistochemical staining of ALP in bone section

A standard protocol was used for the immunohistochemical staining of ALP in bone section according to the manufacturer’s instructions (DAKO Envision^+^ staining kit, Via Real Carpinteria, CA, USA). Antibody against ALP (ab203106, Abcam, Cambridge, CA, USA) was diluted at 1:300. The chromogenic number of ALP-positive osteoblasts was counted per square millimeter (4 × 0.25 mm) paratrabecular with a LEICA DM2000 microscope (× 40 magnification).

### Statistical analysis

Results were analyzed with GraphPad Prism 5.0 software (GraphPad Software Inc., La Jolla, CA, USA). All data are presented as the mean ± standard error of the mean (SEM). An unpaired *t* test was used to compare the mean between two independent groups. A one-way ANOVA was used to compare the means of more than two groups. *P* < 0.05 (*), *P* < 0.01 (**), *P* < 0.001 (***), and *P* < 0.0001 (****) were considered statistically significant.

## Results

### Proteasome inhibitors induce osteogenic differentiation of MSCs and MC3T3-E1 cells

**Fig. 1:**
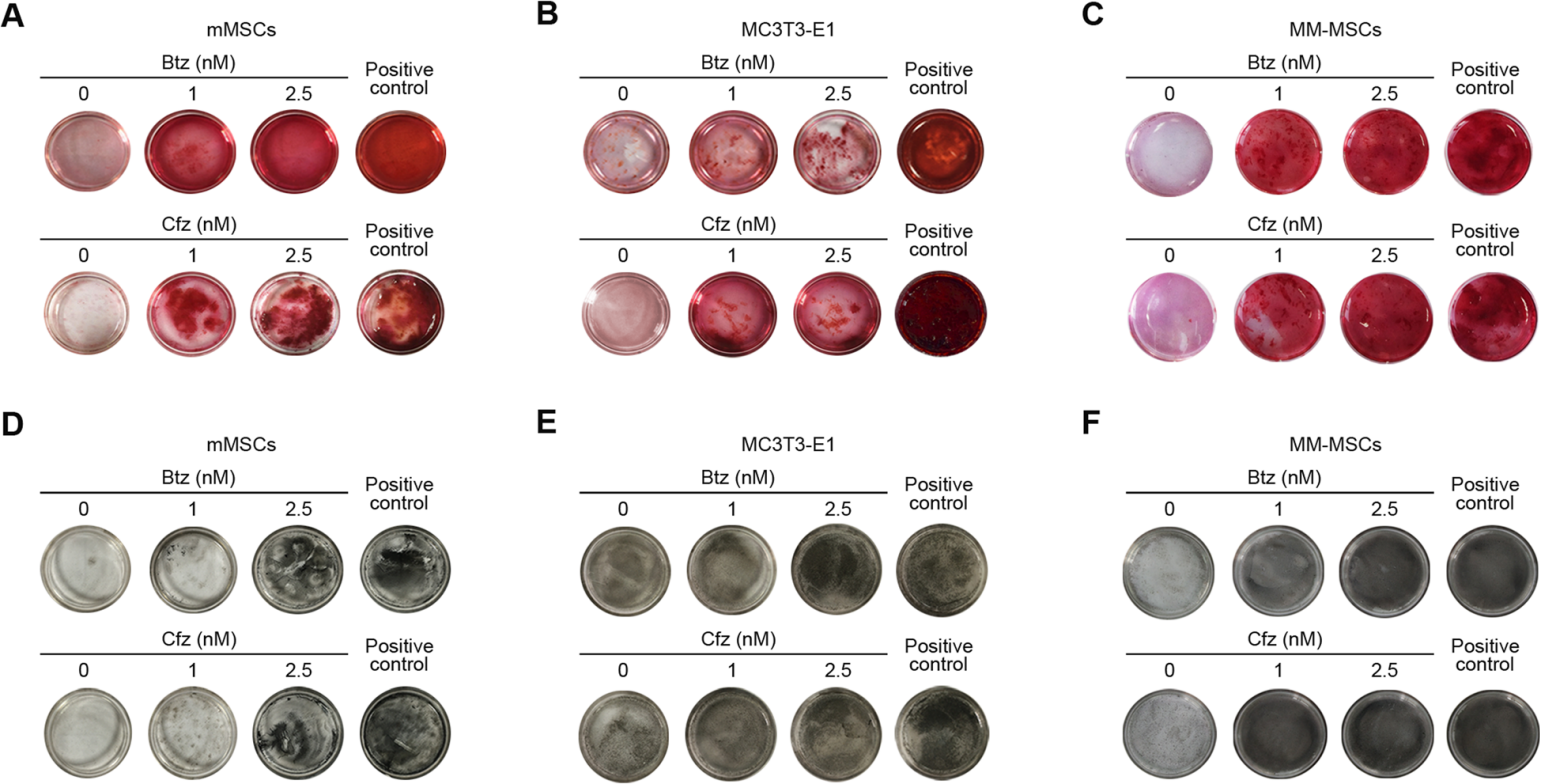
Effects of proteasome inhibitors on osteogenic differentiation of mMSCs, MC3T3-E1 cells, and MM-MSCs. Eighty to 90% confluent mMSCs, MC3T3-E1 cells, and MM-MSCs in 35-mm dishes were treated with bortezomib (Btz) or carfilzomib (Cfz) at concentrations of 0, 1, and 2.5 nM or were cultured in osteogenic differentiation medium for 8 days, replacing with fresh medium every 2 days. Alizarin red staining of mMSCs (**a**), MC3T3-E1 cells (**b**), and MM-MSCs (**c**) treated with bortezomib (upper panel) or carfilzomib (lower panel). Alkaline phosphatase staining of bortezomib (upper panel) or carfilzomib-treated (lower panel) mMSCs (**d**), MC3T3-E1 cells (**e**), and MM-MSCs (**f**). Images shown are representative of 3 independent experiments

To validate that proteasome inhibition-induced osteogenesis is not drug-specific, we compared the effects of two different proteasome inhibitors bortezomib and carfilzomib on osteoblast differentiation by performing ARS and ALP staining. As shown in Fig. 1, we found that low concentrations of bortezomib and carfilzomib significantly and equally induced osteogenic differentiation by increasing calcium deposition and mineralized bone nodule formation after 8 days of treatment. Similar results were also observed in low doses of bortezomib and carfilzomib-treated MM-MSCs and human normal MSCs (Supplemental Figure 1). Given to the structurally and mechanistically difference between bortezomib and carfilzomib, these findings may strongly suggest that targeting proteasome can trigger osteoblast differentiation. Moreover, we found that higher concentrations of PIs are toxic to the cells (Supplemental Figure 2). This is consistent with previous findings that higher doses of bortezomib induce pro-apoptotic signaling [8, 17].

### Proteasome inhibition induces the expression of key osteogenic differentiation-related genes and activates ER stress signaling

**Fig. 2:**
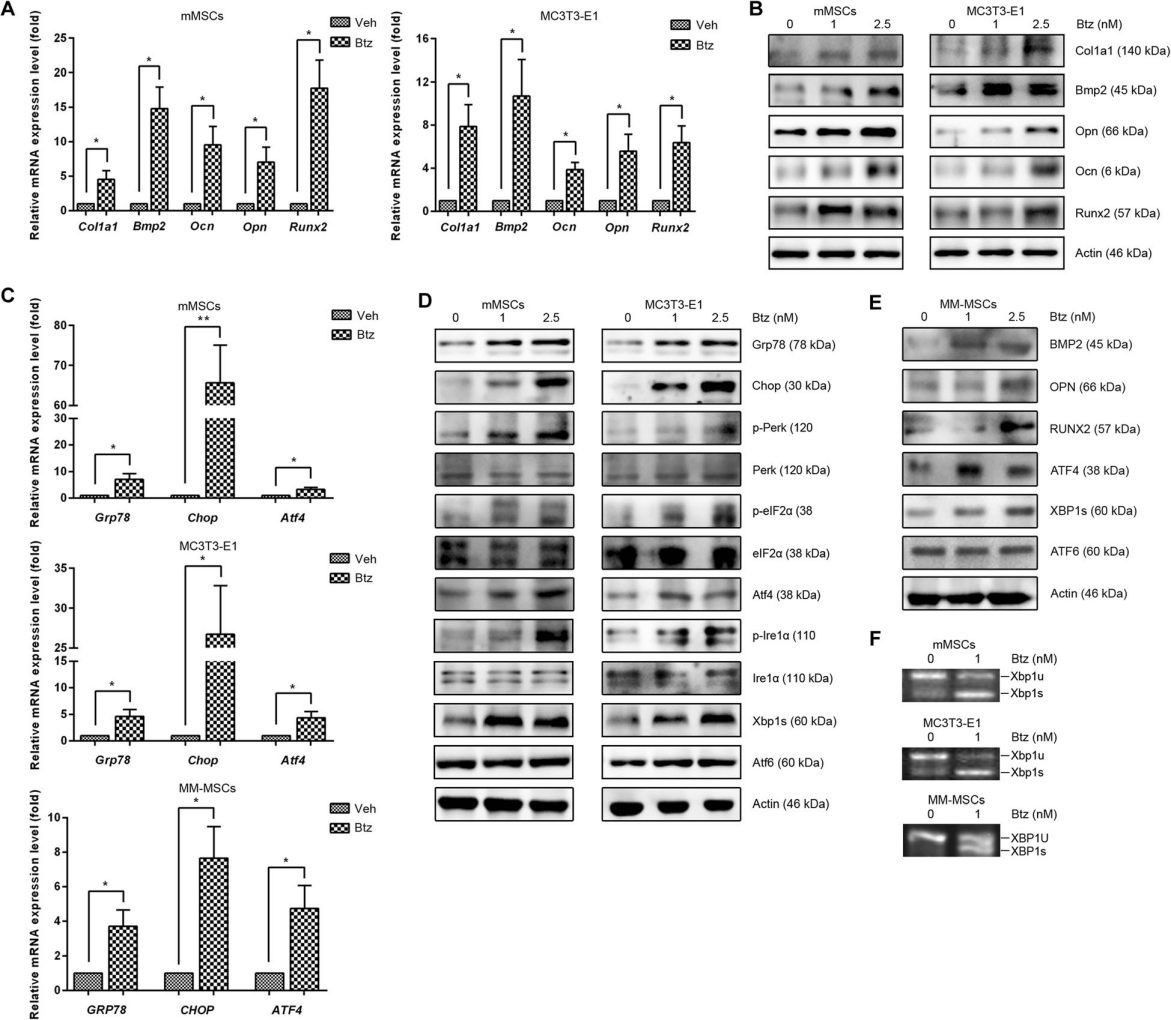
Bortezomib induces the expression of osteoblast differentiation-related markers, and activates ER stress signaling branches. Confluent MSCs or MC3T3-E1 cells were treated with or without bortezomib for 24 h. **a** Real-time PCR analysis of the expression of *Col1a1*, *Ocn*, *Opn*, *Runx2*, and *Bmp2* in 2.5 nM of bortezomib-treated and untreated mMSCs (left panel) or MC3T3-E1 cells (right panel). **b** Western blotting analysis demonstrating the expression of Col1a1, Ocn, Opn, Runx2, and Bmp2 in bortezomib-treated (0, 1, 2.5 nM) mMSCs (left panel) and MC3T3-E1 cells (right panel). **c** Real-time PCR analysis of ER stress markers *Grp78*, *Chop*, and *Atf4* in 2.5 nM of bortezomib-treated and untreated mMSCs (top panel), MC3T3-E1 cells (middle panel), and MM-MSCs (bottom panel). **d** Western blotting analysis of the expression of ER stress signaling in bortezomib-treated (0, 1, 2.5 nM) mMSCs (left panel) and MC3T3-E1 cells (right panel). **e** Western blotting analysis of the changes of the osteogenesis markers and the key components of three major ER stress signaling branches in bortezomib-treated (0, 1, 2.5 nM) MM-MSCs. **f** RT-PCR analysis of the splicing of *Xbp1* in mMSCs (left panel) and MC3T3-E1 cells (right panel) without or with bortezomib-treated (1 nM). Data shown are representative of 3 independent experiments (*P < 0.05, **P < 0.01)

Next, we examined the effects of bortezomib on the expression of osteogenic differentiation-related genes and found that treatment with bortezomib significantly increased the expression of alpha 1 type 1 collagen (*Col1a1*), osteopontin (*OPN*), and osteocalcin (*OCN*), runt-related transcription factor 2 (*RUNX2*), and bone morphogenetic protein 2 (*BMP2*) on the mRNA level (Fig. 2a). Consistent with these observations, we found that bortezomib increased the expression of these osteogenic differentiation-related genes on the protein level (Fig. 2b). These findings indicate that bortezomib can trigger the expression of osteoblastic phenotype-specific genes to induce osteogenic differentiation.

To ascertain whether ER stress signaling is involved in proteasome inhibition-mediated osteoblast differentiation, we further investigated the changes of ER stress markers and the key regulators in response to bortezomib treatment. As shown in Fig. 2c and d, the expression of ER stress markers Grp78, Chop, and Atf4 were significantly upregulated by bortezomib on both mRNA and protein levels, suggesting the activation of ER stress signaling in bortezomib-treated MSCs and MC3T3-E1 cells. Furthermore, among the three major ER stress signaling pathways, we found that bortezomib upregulated the expression levels of Atf4 and Xbp1s, other than Atf6. Focusing on the changes of Atf4, we further found that its upregulation was tightly associated with the enhanced transcription on the mRNA level. Similar changes on osteogenic markers and ER stress regulators were also confirmed in bortezomib-treated MM-MSCs (Fig. 2e) and human MSCs from healthy donors (Supplemental Figure 3). Considering that the changes of Xbp1 are regulated by unconventional mRNA splicing, we used RT-PCR to amplify the expression of unspliced and spliced Xbp1 and demonstrated that bortezomib significantly triggered the splicing of Xbp1 in the three kinds of tested cells (Fig. 2f).

### Both ATF4 and XBP1s ER stress signaling pathways are involved in proteasome inhibition-induced osteogenic differentiation

**Fig. 3:**
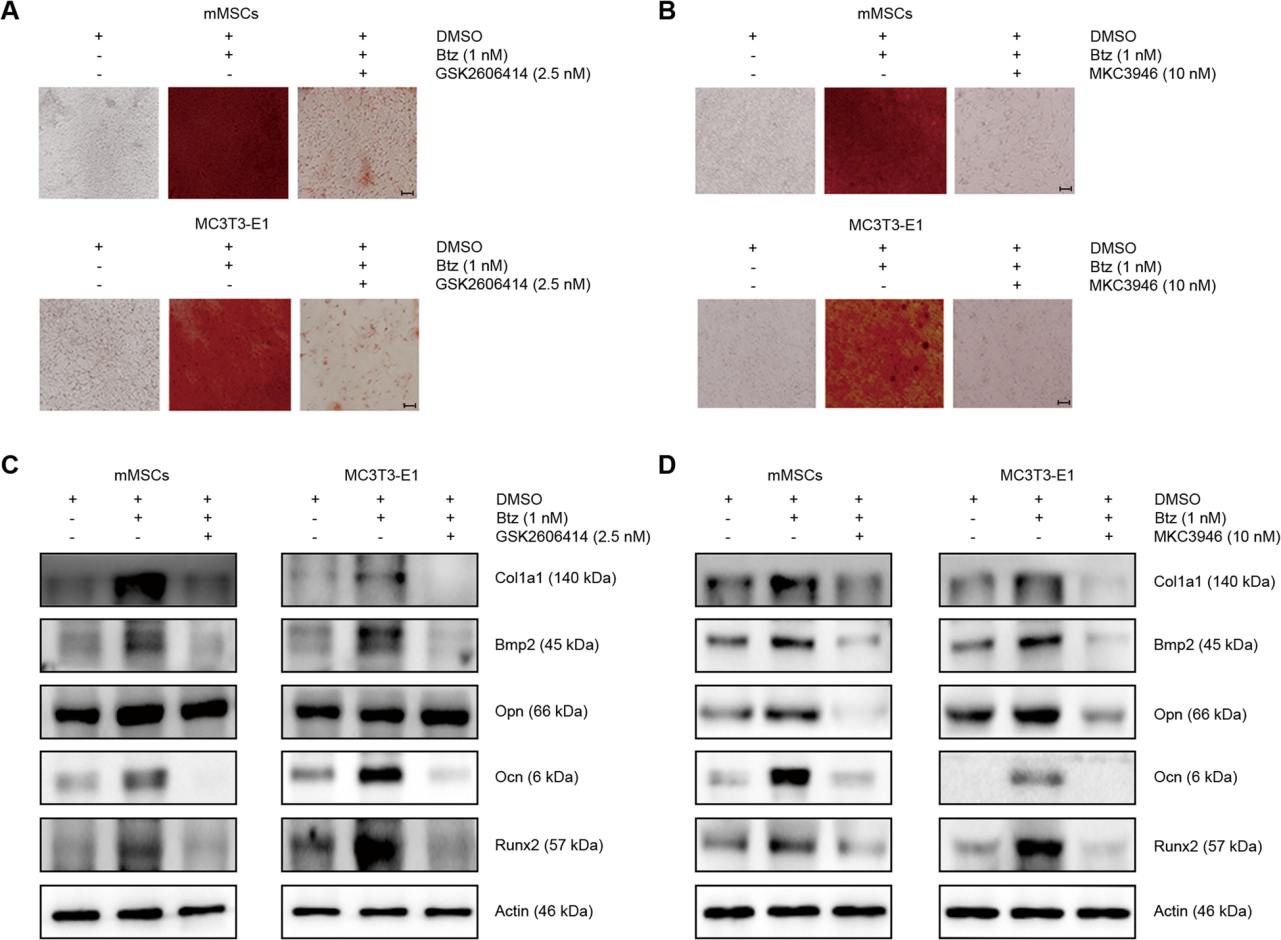
Inhibition of Atf4 and Xbp1s ER stress arms impairs bortezomib-induced osteogenic differentiation. Alizarin red staining shows the effects of GSK2606414 (**a**) or MKC3946 (**b**) on bortezomib-induced osteogenic differentiation in mMSCs (upper panel) and MC3T3-E1 (lower panel) cells. Eighty to 90% confluent MSCs or MC3T3-E1 cells in 35-mm dishes were treated without or with bortezomib (1 nM) in the absence or presence of the PERK inhibitor GSK2606414 (2.5 nM) or IRE1α inhibitor MKC3946 (10 nM) for 8 days. The culture medium was refreshed every 2 days. The images were taken on a Nikon Eclipse TS100 microscope (scale bars represent 50 μM). **c**, **d** Western blotting analysis of the effects of GSK2606414 or MKC3946 on bortezomib-induced osteogenic differentiation-related markers in mMSCs (left panel) and MC3T3-E1 cells (right panel). Confluent MSCs or MC3T3-E1 cells were treated without or with bortezomib (1 nM) in the absence or presence of the PERK inhibitor GSK2606414 (2.5 nM) or IRE1α inhibitor MKC3946 (10 nM) for 24 h. Data shown are representative of 3 independent experiments

To clarify whether the activation of ER stress signaling branch ATF4 and XBP1s are directly involved in bortezomib-induced osteogenic differentiation, we next investigated the underlying mechanisms of each branch in this process. As shown in Fig. 3a and b, we found that the bortezomib-induced bone nodule formation was significantly impaired when combined with a potent PERK inhibitor GSK2606414 or IRE1α inhibitor MKC3946. In addition, we found that the expression of the bortezomib-induced osteogenic differentiation-related marker Col1a1, Ocn, Bmp2, and Runx2 were reversed, whereas the expression of Opn was not changed by GSK2606414 (Fig. 3c). However, when using IRE1α inhibitor MKC3946 to abolish the formation of Xbp1s, we observed that it significantly decreased the expression of all these osteoblastic differentiation-related molecules (Fig. 3d).

### XBP1s regulates the activation of PERK-ATF4 ER stress signaling pathway

**Fig. 4:**
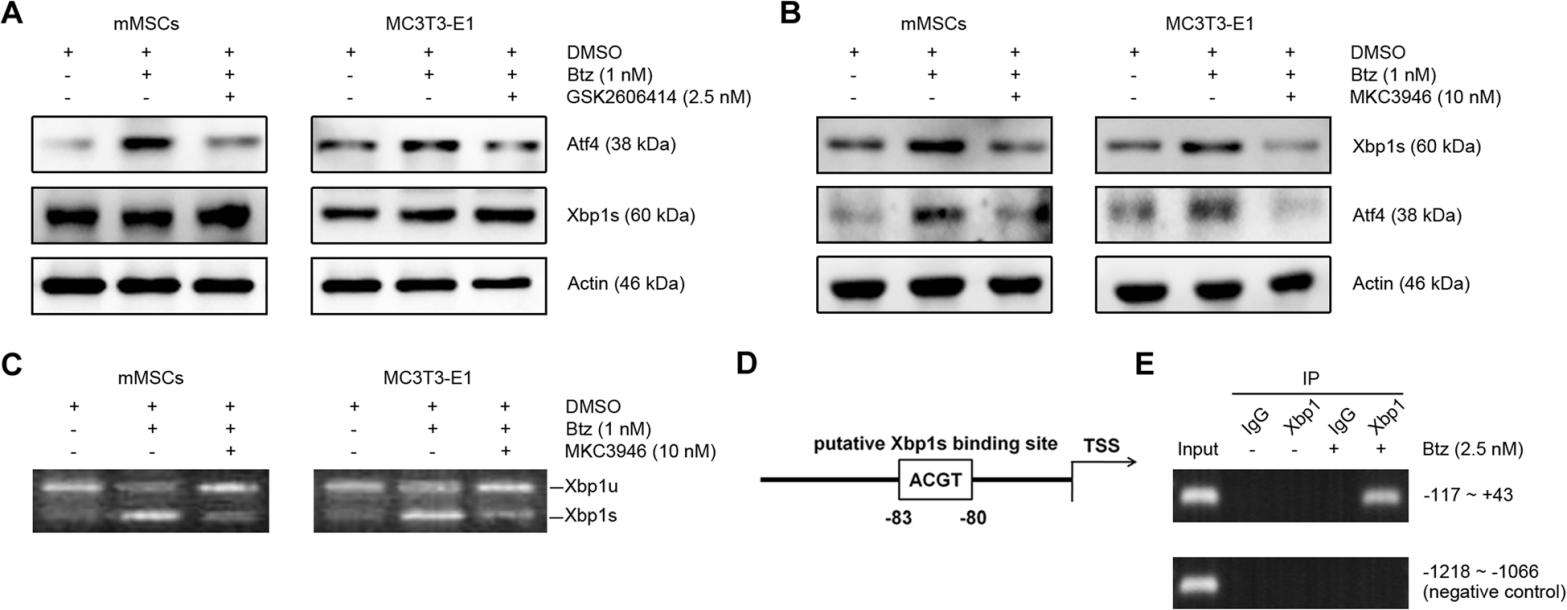
Xbp1s regulates the expression of Atf4. **a**, **b** Western blotting analysis of the effects of GSK2606414 or MKC3946 on bortezomib-activated Atf4 and Xbp1s in mMSCs (left panel) and MC3T3-E1 cells (right panel). Confluent MSCs or MC3T3-E1 cells were treated without or with bortezomib (1 nM) in the absence or presence of the PERK inhibitor GSK2606414 (2.5 nM) or IRE1α inhibitor MKC3946 (10 nM) for 24 h. **c** RT-PCR analysis of the effects of MKC3946 on the bortezomib-induced formation of *Xbp1s* mRNA. MSCs or MC3T3-E1 cells were treated as the abovementioned. **d** A schematic of the promoter region of the *Atf4* gene. One putative Xbp1s-binding site (white rectangle, − 83/− 80) is shown. **e** ChIP assays using control IgG, antibodies against Xbp1s. Cell extracts were collected from mMSCs treated with or without bortezomib (2.5 nM) for 16 h. Protein/DNA complexes from cells were precipitated without antibody (input) or with an Xbp1s antibody or IgG. PCR was performed using a set of primers covering the promoter region containing the putative Xbp1s binding site. PCR using input DNA was used as a positive control. PCR using primers covering the promoter region of the *Atf4* gene (− 1218/− 1066) was used as a negative control. Data shown are representative of 3 independent experiments

To gain further insight into the relationship between XBP1s and ATF4 in bortezomib-induced osteogenic differentiation, we investigated the crosstalk between these two branches. As shown in Fig. 4a, when using PERK inhibitor GSK2606414 to decrease the expression of Atf4, we observed that it did not affect the expression of Xbp1s. However, when using IRE1α inhibitor MKC3946 to inhibit the formation of Xbp1s, we observed that it markedly decreased the expression of Atf4 (Fig. 4b). Moreover, we validated the effect of MKC3946 on decreasing of the bortezomib-induced splicing of Xbp1 by RT-PCR analysis (Fig. 4c). These data strongly suggest that ER stress branch Xbp1s could affect the expression of Atf4. Therefore, we next sought to investigate how Xbp1s regulates the expression of Atf4. Interestingly, nucleotide sequence analysis revealed that it contains 1 binding site for Xbp1s at the promoter region of *Atf4* (Fig. 4d). This finding prompted us to investigate whether Xbp1s acts as a transcription factor for *Atf4*. To test this possibility, we performed ChIP assay using nuclear extracts from bortezomib-treated and untreated mMSCs and a set of primers designed to amplify the promoter region (− 117 to + 43) of *Atf4*. As expected, the PCR amplification of the ChIP assay revealed that Xbp1s was capable of binding to the *Atf4* promoter (Fig. 4e). These observations indicate that the activation of Xbp1s can further transcriptionally enhance the expression of *Atf4* in response to bortezomib treatment.

### Overexpression of human XBP1s triggers osteogenic differentiation

**Fig. 5:**
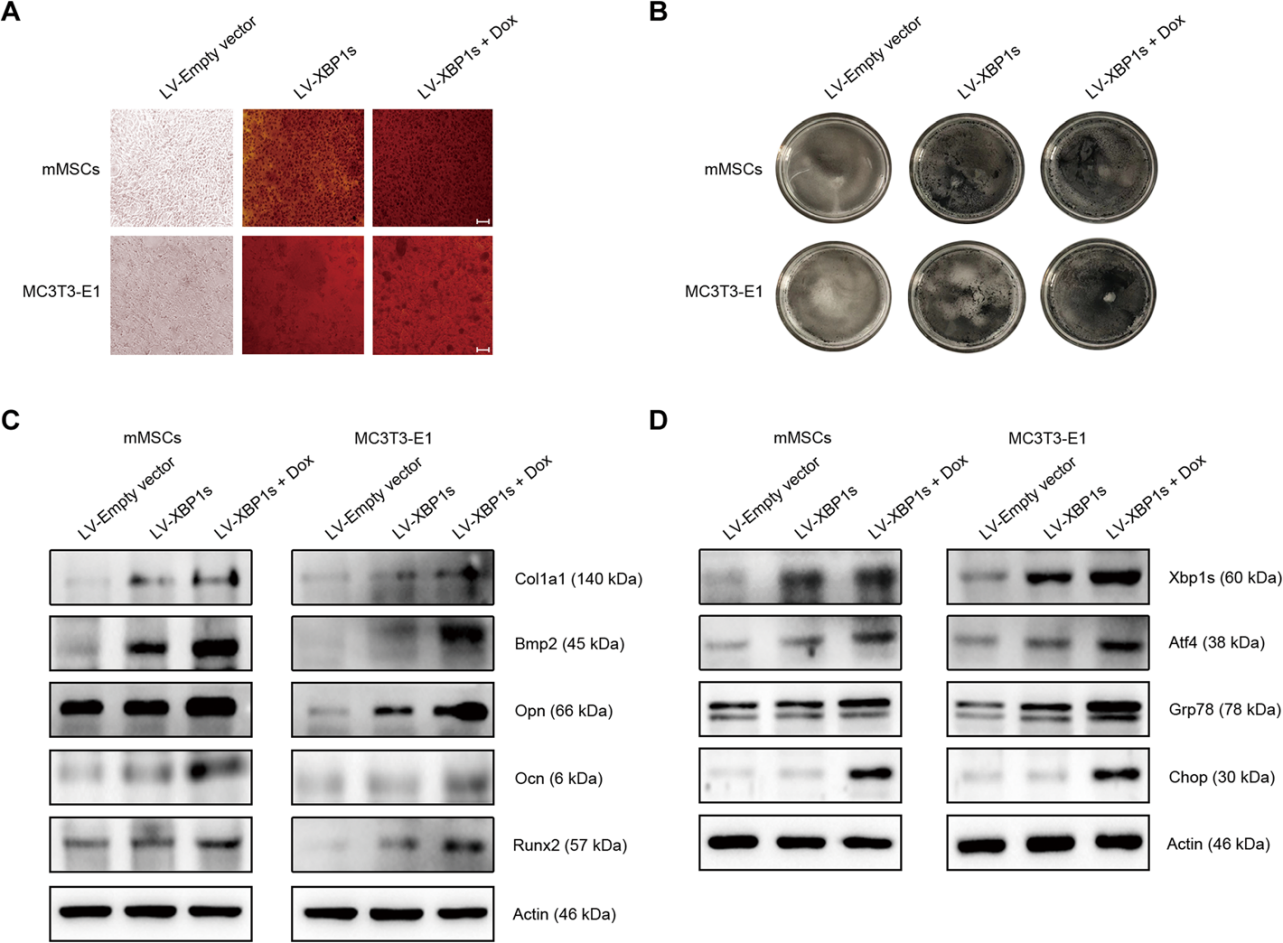
Forced expression of XBP1s induces osteoblast differentiation. mMSCs and MC3T3-E1 cells transduced with lentiviral-XBP1s vectors were cultured in the absence or presence of 2.5 μg/mL of doxycycline to induce a higher level of XBP1s expression. Cells transduced with empty lentiviral vectors were used as control. **a** Alizarin red staining of the mineralized nodule in mMSCs and MC3T3-E1 cells overexpressing XBP1s. The transduced mMSCs and MC3T3-E1 cells were treated with or without doxycycline (Dox) for 5 days and then determined by Alizarin red staining. Images were viewed by a Nikon Eclipse TS100 microscope (scale bars represent 50 μM). **b** Alkaline phosphatase staining of the transduced mMSCs and MC3T3-E1 cells treated with or without doxycycline for 5 days. **c** Western blotting analysis of the osteogenic markers in mMSCs and MC3T3-E1 cells overexpressing XBP1s. mMSCs or MC3T3-E1 cells transduced with lentiviral-XBP1s vectors were cultured in the absence or presence of doxycycline for 24 h. **d** Western blotting analysis of the expression of XBP1s, Atf4, and ER stress markers in the transduced MSCs and MC3T3-E1 cells treated by doxycycline for 24 h. Each assay represents a separate experiment carried out in triplicate

To further validate the key role of Xbp1s in osteogenic differentiation, we used a Tet-On inducible lentiviral vector to overexpress human *XBP1s* in mMSCs and MC3T3-E1 cells. As shown in Fig. 5a, ARS showed that forced expression of XBP1s significantly induced bone nodule formation in mMSCs and MCT3-E1 cells, in contrast to the empty vector-transduced group. Moreover, in these two groups transduced with XBP1s, doxycycline-induced higher level of XBP1s expression was associated with markedly enhanced osteogenic differentiation. Consistent with these findings, we also confirmed higher ALP activity in the two groups overexpressing XBP1s (Fig. 5b). In addition, we found that overexpression of XBP1s could increase the expression of the osteogenic differentiation-related molecules, including Col1a1, Ocn, Bmp2, Runx2, and Opn (Fig. 5c). More importantly, we observed that overexpression of XBP1s induces a higher level of Atf4, as well as Grp78 and Chop (Fig. 5d).

### Inhibition of IRE1α-Xbp1 signaling reverses bortezomib-induced bone formation in mice

**Fig. 6:**
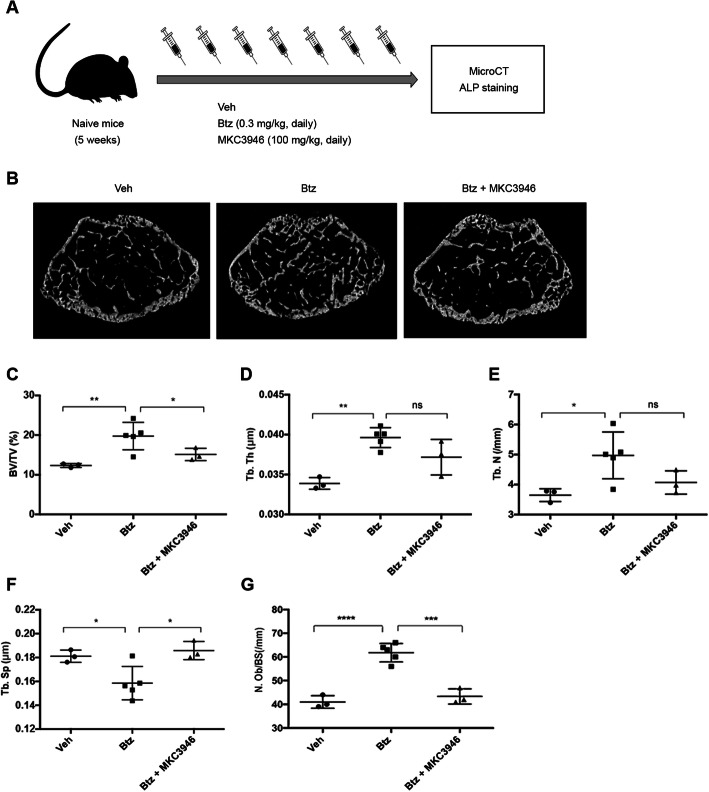
Inhibition of IRE1α reverses bortezomib-increased bone volume and osteoblast number in mice. **a** A schematic diagram showing the treatment protocol. Five-week-old C57BL/KaLwRij mice were treated with vehicle (Veh, *n* = 3), bortezomib (0.3 mg/kg, *n* = 5), and bortezomib combined with MKC3946 (100 mg/kg, *n* = 3) for 7 consecutive days. On day 8, mice were sacrificed, microCT, and immunohistochemical staining used to analyze bone parameters. **b** Three-dimensional reconstructions of microCT scans of the representative femur from vehicle-, bortezomib-, and bortezomib combined with MKC3946-treated naive mice. **c–f** MicroCT analysis of the trabecular bone volume [BV/TV (%)] (**c**), and trabecular thickness [Tb.Th (/mm^2^)] (**d**), trabecular number [Tb.N (/mm)] (**e**), and trabecular separation [Tb.Sp (μm)] (**f**) of the femur of mice treated with vehicle, bortezomib, and bortezomib combined with MKC3946. **g** Histograms showing osteoblast number [Ob.N/BS (/mm)] in mice treated with vehicle, bortezomib, and bortezomib combined with MKC3946. Data represent mean ± SEM; ns, no significance; *P < 0.05, **P < 0.01, ***P < 0.001, ****P < 0.0001

To further determine the indispensable role of Xbp1s in proteasome inhibition-induced osteoblast differentiation in vivo, we investigated the effects on new bone formation in young mice treated with bortezomib in the presence or absence of the IRE1α inhibitor MKC3946 (Fig. 6a). We demonstrated that bortezomib enhanced the bone formation in the femur of the treated mice. Nonetheless, inhibition Xbp1s formation with IRE1α inhibitor MKC3946 significantly abolished bortezomib-mediated bone formation (Fig. 6b). Parametric analysis using microCT revealed a significant increase in the bone volume (bone volume/tissue volume), trabecular thickness, and trabecular number by bortezomib (Fig. 6c–e). In contrast, the trabecular separation was decreased by bortezomib (Fig. 6f). However, when using MKC3946 to inhibit Xbp1s signaling in mouse, we observed that bortezomib-enhanced bone volume was significantly decreased to the level of the vehicle-treated control group. Similarly, both trabecular thickness and trabecular number were also decreased by MKC3946, but no statistically significant difference was observed. Simultaneously, the bortezomib-decreased trabecular separation was increased to the level of the vehicle-treated control group. Importantly, we observed similar changes in the number of osteoblasts in the femur by the combinational treatment with bortezomib and MKC3946 (Fig. 6g). These data further support that bortezomib can increase bone formation through activation of the XBP1s pathway in vivo.

## Discussion

In this study, we provided both in vitro and in vivo evidence indicating that ER stress sensor XBP1s is a master regulator of the PI-induced bone formation. PIs can activate XBP1s in MSCs, which further drives the osteogenic differentiation, and therefore promotes new bone formation.

As a common and devastating complication of MM, MBD is characterized by osteolytic destruction that results from uncoupled or severely imbalanced bone remodeling with increased osteoclast activity and suppresses osteoblast function [2]. Currently, bisphosphonates are the only pharmacological agents recommended for the treatment and prevention of MBD [18]. As pyrophosphate analogs, bisphosphonates bind to hydroxyapatite crystals and are incorporated into the exposed bone areas. During bone remodeling, bisphosphonates are absorbed by osteoclasts and lead to inhibition of osteoclast activity and the induction of apoptosis. Both preclinical and clinical data have demonstrated that PIs also show a striking bone protection benefit in patients with MM. Due to the high apoptotic sensitivity of MM cells to proteasome inhibition, PI-induced bone protection is considered to be associated with the reduced tumor burden and the inhibited osteoclast formation and activity [9, 19, 20]. Nonetheless, emerging data also indicate that proteasome inhibition may play a critical role in the regulation of osteoblast differentiation. Several different groups have demonstrated that PI-induced bone protection is tightly related to the increased number or activity of osteoblasts in MM [8, 9, 21–23], but the underlying molecular mechanisms remain poorly understood.

The ubiquitin-proteasome system (UPS) is a key regulator of intracellular protein hemostasis, cell cycle control, metabolism, survival, apoptosis, and elimination of damaged proteins that are toxic to the cells. The function of MM cells is more dependent on UPS to degrade the unfolded/misfolded proteins and functional proteins accumulated in the ER lumen and cytosol, since it contains a high amount of secreted proteins, including monoclonal immunoglobulin and various cytokines. However, when inhibiting the UPS pathway, the accumulation of the unfolded/misfolded proteins and functional proteins in the ER lumen and cytosol will trigger ER stress and the subsequent ER stress-induced apoptosis or resistance [15, 24, 25]. Recent studies revealed that the mechanisms of action of proteasome inhibition in MM therapy were related to the activation of ER stress. ER stress-triggered pro-apoptotic signaling has emerged gradually to be the main mechanism of inducing MM cell death by PIs, in addition to the inhibition of NF-κB signaling [13, 24, 26]. Given the importance of ER in maintaining protein, lipid, and redox homeostasis, controlling secretory processes, ER stress is an essential regulatory mechanism that is aimed at allowing cells to adapt to the changing environment. Correspondingly, ER stress initiates an intrinsic signaling network, which consists of PERK-ATF4, IRE1α-XBP1, and ATF6, three main pathways that lead to major changes in transcriptional programs. As such, ER stress has been implicated in many aspects of cellular function and dysfunction, including survival, proliferation, autophagy, migration, and differentiation [27, 28].

**Fig. 7:**
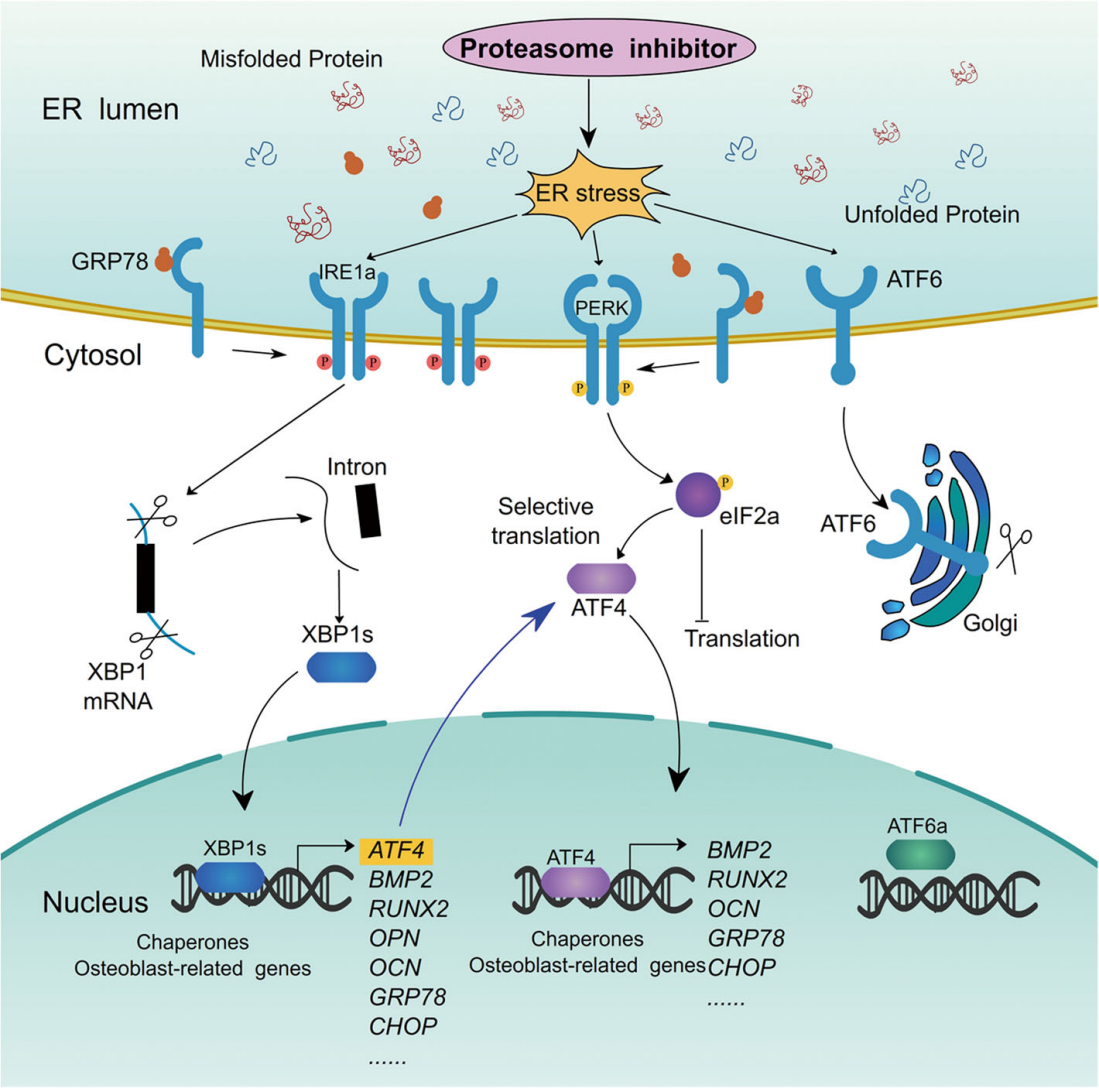
Schematic diagram depicting the roles of ER stress signaling arm Xbp1s in regulating osteoblast differentiation induced by proteasome inhibitors

We speculated that ER stress might be an important regulator of the bone protection induced by proteasome inhibitors in MM (Fig. 7). In this regard, Garrett et al. initially found the link between the UPS and bone remodeling and showed that selective inhibition of proteasome stimulates bone formation in mouse [6]. Thereafter, it was further demonstrated by Mukherjee et al. that MSCs are the preferential stem/progenitor population that were induced by bortezomib to differentiate into osteoblasts [29]. In MM, it was further demonstrated that bortezomib can promote osteoblast activity in vitro and in vivo [8, 21, 22]. Along these lines, we further revealed that targeting proteasome can activate ER stress signaling in MSCs ongoing osteogenesis. Importantly, we found that the activation of both ATF4 and XBP1s branches were involved in the regulation of PI-induced osteoblast differentiation. In terms of the ATF4, it has been implicated in the regulation of osteoblast biology [30–33]. ATF4 was initially found to transcriptionally regulate the expression of *Ocn*, which acts in the bone matrix to regulate mineralization [34]. ATF4 was also demonstrated to be a critical determinant of bone mass postnatally and toughness through posttranscriptional mechanisms by upregulating the synthesis of *Col1a1* [31, 32]. As a transcription factor, ATF4 can be induced by a variety of stress stimuli to regulate osteoblast differentiation [30, 32, 33, 35–41]. Concerning the relationship between ATF4 and proteasome inhibition, it has been suggested that bortezomib can activate ATF4 to affect osteoblast differentiation [11, 17]. In agreement with previously published data, herein, we confirmed that proteasome inhibition can regulate osteogenic differentiation through activating ATF4, and further demonstrated that proteasome inhibition-induced ATF4 can affect the expression of osteoblast-specific genes and the osteoblastic mineralization.

One of the important findings in this study may be the key role of XBP1s arm of ER stress in proteasome inhibition-induced osteoblast differentiation. Upon the induction of ER stress, the activation of the endonuclease activity of IRE1α by dimerization and autophosphorylation leads to unconventional splicing of *XBP1* mRNA, which will be translated to be an active transcription factor XBP1s [42]. Our observations indicate that XBP1s arm was activated in response to proteasome inhibition stimuli on both mRNA and protein levels. Moreover, when using the IRE1α inhibitor to inactivate this signaling pathway, we discovered that the reduced Xbp1s formation strikingly impaired bortezomib-induced mineralization and significantly reduced the expression of osteogenic differentiation markers in MM-MSCs (Supplemental Figure 4). In contrast, inhibition of the ATF4 arm caused nearly but not completely the osteoblastic mineralization. In addition, we confirmed that inhibition of Xbp1s can downregulate the expression of Atf4; conversely, inhibition of ATF4 arm had no effects on the expression of Xbp1s. In support of this finding, we overexpressed human XBP1s in mouse MSCs and demonstrated that XBP1s can trigger osteogenic differentiation and bone mineralization. Consistently, we confirmed an increased expression of Atf4 in this setting. These data strongly indicate a feedback loop that Xbp1s positively upregulates the expression of Atf4. To further investigate the regulatory mechanism between Xbp1s and Atf4, we performed a ChIP assay and identified a Xbp1s binding motif at the promoter region of the *Atf4* gene. Limited by the difficulty of obtaining MM-MSCs, the above findings are mainly based on normal mMSCs and MC3T3-E1 cells. Given to the demonstrated difference of MM-MSCs from their healthy counterparts and the potential effects of tumor microenvironment, it should be very important to further validate the findings in murine MM model in future.

Transcription factor XBP1s is highly conserved and has been shown to govern various cell-fate decisions, including differentiation, metabolism, apoptosis, and drug resistance [42–46]. Previous studies have linked XBP1s to adipogenesis, osteoclastogenesis, eosinophil, and plasma cell differentiation [44, 47–49]. It has been shown that XBP1s is tightly related to BMP2-induced osteoblast differentiation of mouse embryonic fibroblasts through promoting the transcription of *Osterix* [50], which is indispensable for bone formation. In the current study, our in vitro results strongly support a pivotal role of XBP1s in proteasome inhibition-induced osteoblast differentiation of MSCs. To confirm this finding in vivo, we further investigated the role of XBP1s in regulating bone formation in a mouse model. Histomorphometry revealed that the trabecular bone volume as a proportion of tissue volume (BV/TV, %), trabecular thickness (Tb.Th, /mm^2^), and trabecular number (Tb.N, /mm) were significantly increased in the femurs of bortezomib-treated young mice. Similarly, the number of osteoblasts in femurs from bortezomib-treated mice is also significantly higher than the control group (Supplemental Figure 5). On the contrary, when the IRE1α inhibitor MKC3946 was combined with bortezomib, as expected, it reversed the bone structure parameters like BV/TV and the numbers of osteoblast. These findings further reveal that ER stress signaling XBP1s is an essential regulator of osteoblast differentiation in vivo.

## Conclusion

In conclusion, the present study revealed that the activation of ER stress signaling pathway IRE1α-XBP1 is a pivotal mediator of proteasome inhibition-induced osteoblast differentiation. The importance of XBP1s in proteasome inhibition-induced bone formation relies on its direct effects on the transcription of osteogenic differentiation-related genes, and the positive feedback control of the expression of another ER stress arm ATF4. Our findings provide evidence for a better understanding of the mechanisms of PI-induced bone protection in MM and provide the impetus for future studies to investigate XBP1s potential as a target for the prevention and treatment of MBD.

## Supplementary Information

**Additional file 1: Supplemental Figure 1.** Effects of proteasome inhibitors on osteogenic differentiation of human MSCs. Human bone marrow mesenchymal stem cells (hMSCs) were obtained from the femoral head of patients undergoing hip replacement surgery for femoral head osteonecrosis with informed consent, following a protocol previously described by De Becker A, et al. (*Haematologica* 2007; 92(4): 440–449). 80%–90% confluent hMSCs in 35 mm dishes were treated with bortezomib (Btz) or carfilzomib (Cfz) at concentrations of 0, 1, 2.5 nM, or were cultured in osteogenic differentiation medium for 8 days, and the medium was replaced every 2 days. (**A**) Alizarin red staining of hMSCs treated with bortezomib (upper panel) or carfilzomib (lower panel). (**B**) Alkaline phosphatase staining of bortezomib (upper panel) or carfilzomib-treated (lower panel) hMSCs. Images shown are representative of 3 independent experiments.

**Additional file 2: Supplemental Figure 2.** Flow cytometry analysis of the effects of bortezomib on cell apoptosis. mMSCs and MC3T3-E1 cells were treated with various concentrations of bortezomib for 24 h, then stained with Annexin V-FITC/7-AAD Apoptosis Detection Kit (#640922, Biolegend, CA, USA). The samples were analyzed using a BD FACSCanto II flow cytometer and FlowJo software package V7.6.1 (Tree Star, Inc., OR, USA). Data are representative of three independent experiments.

**Additional file 3: Supplemental Figure 3.** Western blotting analysis of the expression of XBP1s, ATF4, ATF6 and the osteogenic differentiation markers in bortezomib-treated hMSCs. Confluent hMSCs were treated with bortezomib (0, 1, 2.5 nM) for 24 h, the cell lysates were then harvested for Western blotting analysis.

**Additional file 4: Supplemental Figure 4.** Realtime PCR analysis of the expression of osteogenesis markers in MM-MSCs. Confluent MM-MSCs were treated with vehicle (Veh), MKC3946 (10 nM), bortezomib (Btz, 2.5 nM), and the combination for 24 h, then mRNA was extracted and Realtime PCR was performed with primers *COL1A1*, *BMP2*, *OCN* (*Osteocalcin*), *OPN* (*Osteopontin*), and *RUNX2*. Data represent mean ± SEM, ns: no significance, * *P* < 0.05.

**Additional file 5: Supplemental Figure 5.** Immunohistochemical staining of osteogenesisi marker ALP in mouse femur bone paraffin-embedded sections. (A-C) Representative photomicrographs of the IHC staining for ALP in the femur bone section from mice treated with vehicle (A), bortezomib (B), or bortezomib combined with MKC3946. Arrows indicate osteobalsts. Scale bars represent 125 μM.

**Additional file 6: Supplemental Method 1.**

**Additional file 7: Supplemental Table 1.** List of antibodies used for Western blotting.

**Additional file 8: Supplemental Table 2.** Primer sequences used for Realtime PCR analysis.

**Additional file 9: Supplemental Table 3.** Primer sequences used for ChIP assay.

## Data Availability

The data used to support the findings of this study are available from the corresponding author upon request.

## Abbreviations

ALP: Alkaline phosphatase
ARS: Alizarin red S
ATF4: Activating transcription factor 4
ATF6: Activating transcription factor 6
BMP2: Bone morphogenetic protein 2
BV: Bone volume
ChIP: Chromatin immunoprecipitation
Col1a1: Alpha 1 type 1 collagen
DMEM: Dulbecco’s modified Eagle’s medium
ER: Endoplasmic reticular
IRE1α: Inositol-requiring protein 1α
MBD: Myeloma bone disease
MM: Multiple myeloma
MM-MSCs: MSCs derived from MM patients
mMSCs: Mouse bone marrow mesenchymal stem cells
MSCs: Mesenchymal stem cells
OCN: Osteocalcin
OPN: Osteopontin
PERK: PKR-like ER kinase
PIs: Proteasome inhibitors
PVDF: Polyvinylidene difluoride
RIPA: Radioimmunoprecipitation assay
RUNX2: Runt-related transcription factor 2
SDS-PAGE: Sodium dodecyl sulfate-polyacrylamide gel electrophoresis
Tb.N: Trabecular number
Tb.Th: Trabecular thickness
TV: Tissue volume
UPS: Ubiquitin-proteasome system
XBP1: X box binding protein 1
