# Supplementary material for: ER stress arm XBP1s plays a pivotal role in proteasome inhibition-induced bone formation
Source: Stem Cell Res Ther. 2020 Nov 30;11:516. doi: 10.1186/s13287-020-02037-3 (PMC7708206; doi:10.1186/s13287-020-02037-3)
Supplement: Supplementary file 1 — Additional file 1: Supplemental Figure 1. Effects of proteasome inhibitors on osteogenic differentiation of human MSCs. Human bone marrow mesenchymal stem cells (hMSCs) were obtained from the femoral head of patients undergoing hip replacement surgery for femoral head osteonecrosis with informed consent, following a protocol previously described by De Becker A, et al. (Haematologica 2007; 92(4): 440–449). 80%–90% confluent hMSCs in 35 mm dishes were treated with bortezomib (Btz) or carfilzomib (Cfz) at concentrations of 0, 1, 2.5 nM, or were cultured in osteogenic differentiation medium for 8 days, and the medium was replaced every 2 days. (A) Alizarin red staining of hMSCs treated with bortezomib (upper panel) or carfilzomib (lower panel). (B) Alkaline phosphatase staining of bortezomib (upper panel) or carfilzomib-treated (lower panel) hMSCs. Images shown are representative of 3 independent experiments. [file 13287_2020_2037_MOESM1_ESM.docx]

**Supplemental Figure 1**

**
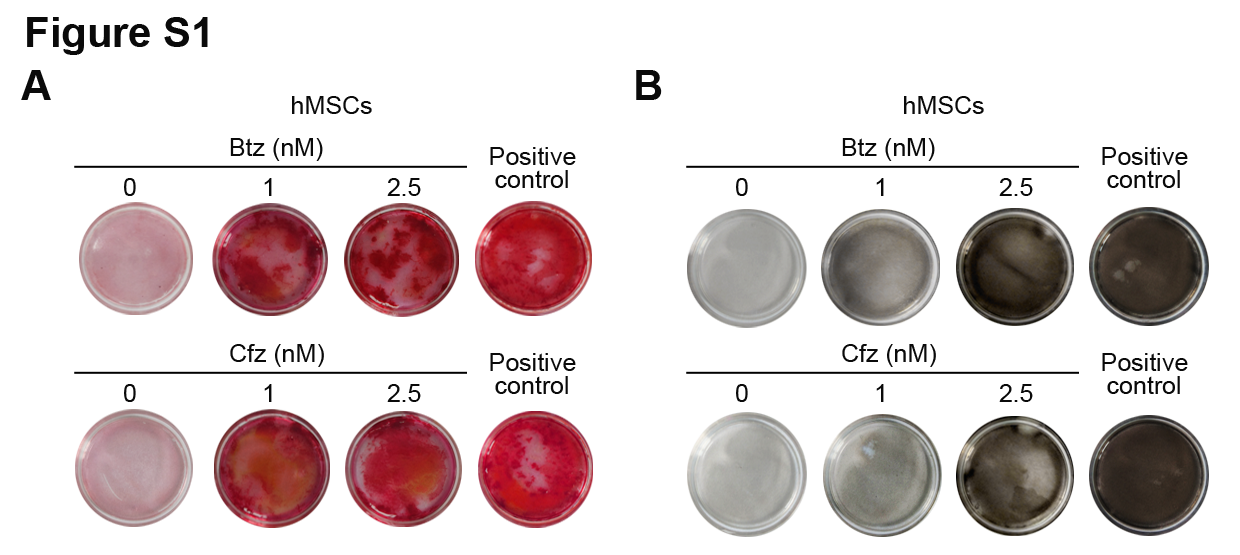
**

**Supplemental Figure 1. Effects of proteasome inhibitors on osteogenic differentiation of human MSCs.** Human bone marrow mesenchymal stem cells (hMSCs) were obtained from the femoral head of patients undergoing hip replacement surgery for femoral head osteonecrosis with informed consent, following a protocol previously described by De Becker A, *et al*. (*Haematologica* 2007; 92(4): 440-449). 80%-90% confluent hMSCs in 35mm dishes were treated with bortezomib (Btz) or carfilzomib (Cfz) at concentrations of 0, 1, 2.5 nM, or were cultured in osteogenic differentiation medium for 8 days, and the medium was replaced every 2 days. (**A**) Alizarin red staining of hMSCs treated with bortezomib (upper panel) or carfilzomib (lower panel). (**B**) Alkaline phosphatase staining of bortezomib (upper panel) or carfilzomib-treated (lower panel) hMSCs. Images shown are representative of 3 independent experiments.
