# Supplementary material for: ER stress arm XBP1s plays a pivotal role in proteasome inhibition-induced bone formation
Source: Stem Cell Res Ther. 2020 Nov 30;11:516. doi: 10.1186/s13287-020-02037-3 (PMC7708206; doi:10.1186/s13287-020-02037-3)
Supplement: Supplementary file 2 — Additional file 2: Supplemental Figure 2. Flow cytometry analysis of the effects of bortezomib on cell apoptosis. mMSCs and MC3T3-E1 cells were treated with various concentrations of bortezomib for 24 h, then stained with Annexin V-FITC/7-AAD Apoptosis Detection Kit (#640922, Biolegend, CA, USA). The samples were analyzed using a BD FACSCanto II flow cytometer and FlowJo software package V7.6.1 (Tree Star, Inc., OR, USA). Data are representative of three independent experiments. [file 13287_2020_2037_MOESM2_ESM.docx]

**Supplemental Figure 2**

**
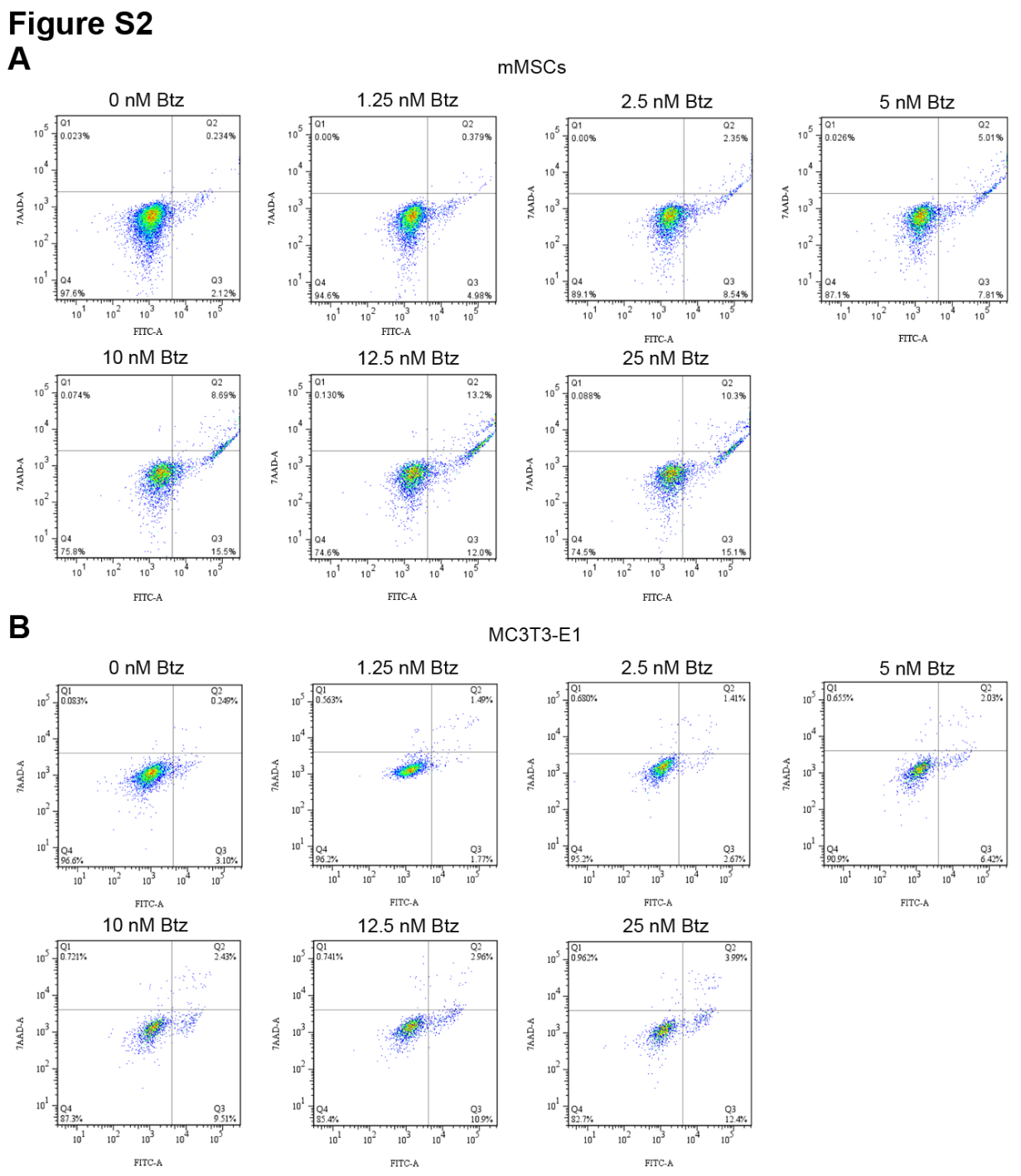
**

**Supplemental Figure 2. Flow cytometry analysis of the effects of bortezomib on cell apoptosis.** mMSCs and MC3T3-E1 cells were treated with various concentrations of bortezomib for 24 h, then stained with Annexin V-FITC/7-AAD Apoptosis Detection Kit (#640922, Biolegend, CA, USA). The samples were analyzed using a BD FACSCanto II flow cytometer and FlowJo software package V7.6.1 (Tree Star, Inc., OR, USA). Data are representative of three independent experiments.
