# Supplementary material for: ER stress arm XBP1s plays a pivotal role in proteasome inhibition-induced bone formation
Source: Stem Cell Res Ther. 2020 Nov 30;11:516. doi: 10.1186/s13287-020-02037-3 (PMC7708206; doi:10.1186/s13287-020-02037-3)
Supplement: Supplementary file 3 — Additional file 3: Supplemental Figure 3. Western blotting analysis of the expression of XBP1s, ATF4, ATF6 and the osteogenic differentiation markers in bortezomib-treated hMSCs. Confluent hMSCs were treated with bortezomib (0, 1, 2.5 nM) for 24 h, the cell lysates were then harvested for Western blotting analysis. [file 13287_2020_2037_MOESM3_ESM.docx]

**Supplemental Figure 3**

**
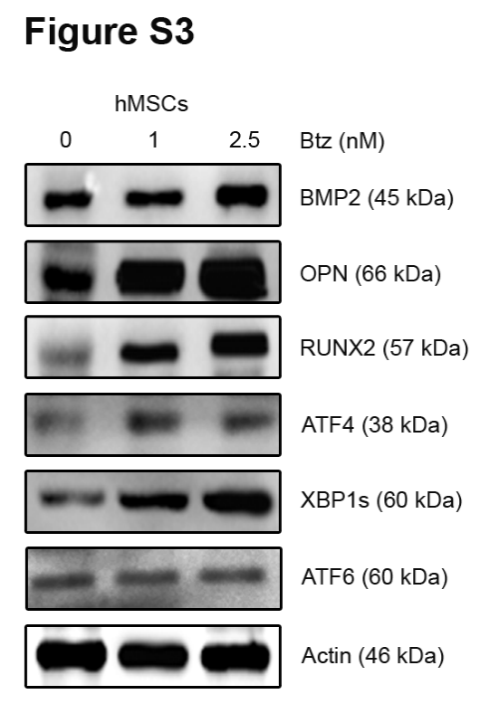
**

**Supplemental Figure 3. Western blotting analysis of the expression of XBP1s, ATF4, ATF6 and the osteogenic differentiation markers in bortezomib-treated hMSCs.** Confluent hMSCs were treated with bortezomib (0, 1, 2.5 nM) for 24 h, the cell lysates were then harvested for Western blotting analysis.
