# Supplementary material for: ER stress arm XBP1s plays a pivotal role in proteasome inhibition-induced bone formation
Source: Stem Cell Res Ther. 2020 Nov 30;11:516. doi: 10.1186/s13287-020-02037-3 (PMC7708206; doi:10.1186/s13287-020-02037-3)
Supplement: Supplementary file 4 — Additional file 4: Supplemental Figure 4. Realtime PCR analysis of the expression of osteogenesis markers in MM-MSCs. Confluent MM-MSCs were treated with vehicle (Veh), MKC3946 (10 nM), bortezomib (Btz, 2.5 nM), and the combination for 24 h, then mRNA was extracted and Realtime PCR was performed with primers COL1A1, BMP2, OCN (Osteocalcin), OPN (Osteopontin), and RUNX2. Data represent mean ± SEM, ns: no significance, * P < 0.05. [file 13287_2020_2037_MOESM4_ESM.docx]

**Supplemental Figure 4**

**
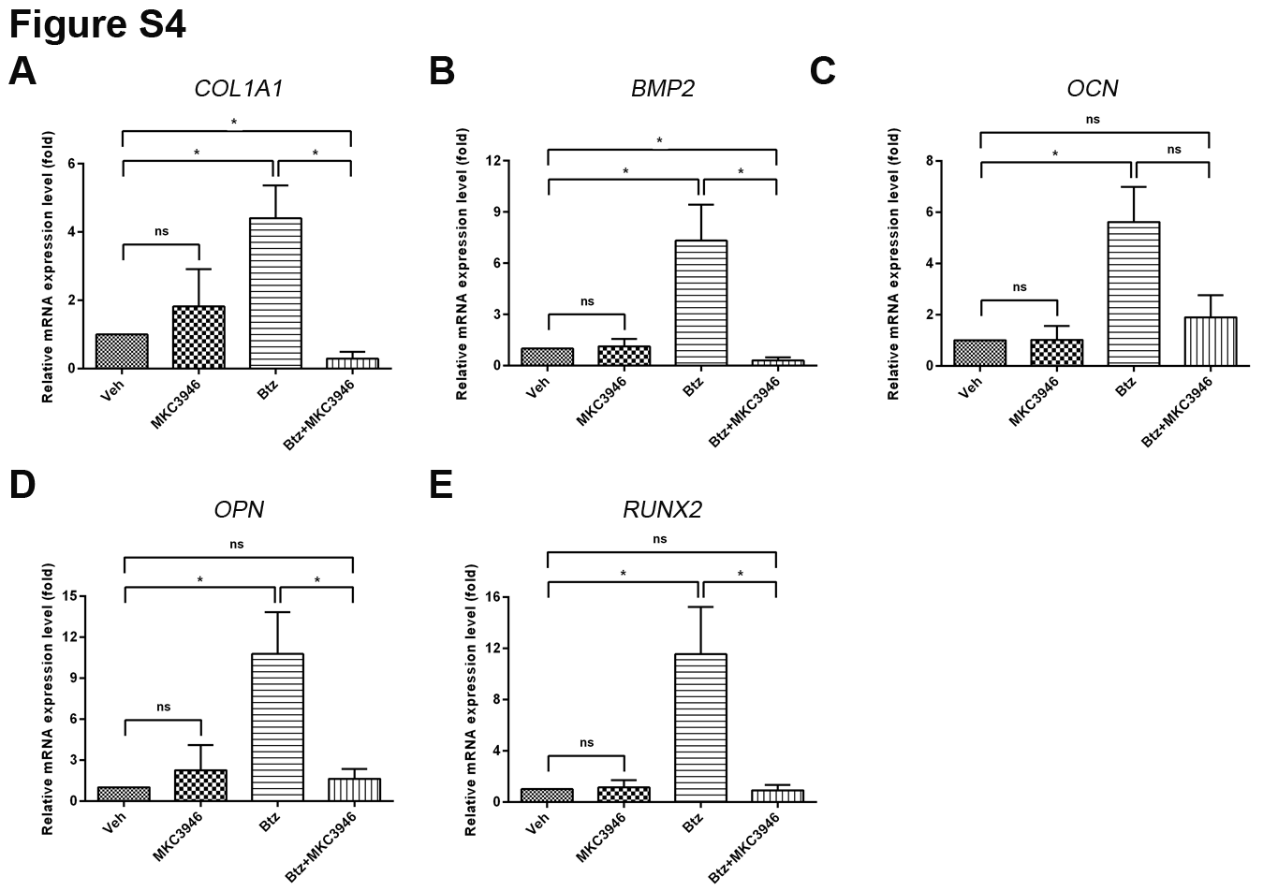
**

**Supplemental Figure 4. Realtime PCR analysis of the expression of osteogenesis markers in MM-MSCs.** Confluent MM-MSCs were treated with vehicle (Veh), MKC3946 (10 nM), bortezomib (Btz, 2.5 nM), and the combination for 24 h, then mRNA was extracted and Realtime PCR was performed with primers *COL1A1*, *BMP2*, *OCN (Osteocalcin)*, *OPN (Osteopontin)*, and *RUNX2*. Data represent mean ± SEM, ns: no significance, * *P* < 0.05.
