# Supplementary material for: ER stress arm XBP1s plays a pivotal role in proteasome inhibition-induced bone formation
Source: Stem Cell Res Ther. 2020 Nov 30;11:516. doi: 10.1186/s13287-020-02037-3 (PMC7708206; doi:10.1186/s13287-020-02037-3)
Supplement: Supplementary file 5 — Additional file 5: Supplemental Figure 5. Immunohistochemical staining of osteogenesisi marker ALP in mouse femur bone paraffin-embedded sections. (A-C) Representative photomicrographs of the IHC staining for ALP in the femur bone section from mice treated with vehicle (A), bortezomib (B), or bortezomib combined with MKC3946. Arrows indicate osteobalsts. Scale bars represent 125 μM. [file 13287_2020_2037_MOESM5_ESM.docx]

**Supplemental Figure 5**


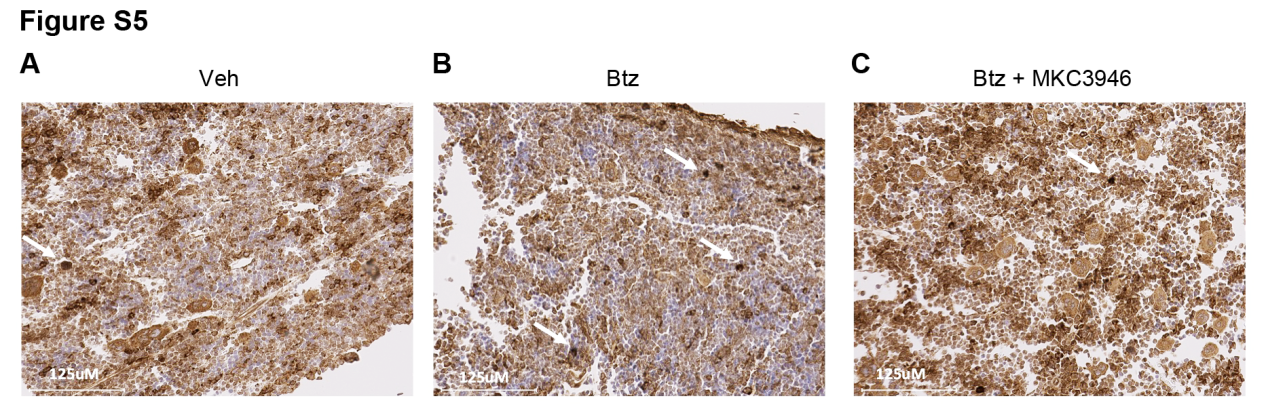


**Supplemental Figure 5. Immunohistochemical staining of osteogenesisi marker ALP in mouse femur bone paraffin-embedded sections. (A-C)** Representative photomicrographs of the IHC staining for ALP in the femur bone section from mice treated with vehicle (A), bortezomib (B), or bortezomib combined with MKC3946. Arrows indicate osteobalsts. Scale bars represent 125 µM.
