# Supplementary material for: ER stress arm XBP1s plays a pivotal role in proteasome inhibition-induced bone formation
Source: Stem Cell Res Ther. 2020 Nov 30;11:516. doi: 10.1186/s13287-020-02037-3 (PMC7708206; doi:10.1186/s13287-020-02037-3)
Supplement: Supplementary file 6 — Additional file 6: Supplemental Method 1. [file 13287_2020_2037_MOESM6_ESM.docx]

**Supplemental Method 1**

**Isolation and expansion of mouse bone marrow MSCs**

The 4-6 week-old C57BL/6 mice were sacrificed by cervical dislocation, and the whole body was soaked thoroughly with 70% ethanol solution for 2 min. The hind legs and vertebrae were dissected, and all tissues around the bones were removed then the bones were placed in a Petri dish with 5 mL of high glucose Dulbecco’s modified Eagle’s medium (DMEM) (BasalMedia, Shanghai, China). The ligaments were cut between the femur and hip, and below the ankle joint. The separation of the tibia from the femur was achieved by bending slightly at the knee joint. The femur/tibia was held with sterile forceps, and then both epiphyses were removed by using sterile scissors. Bone marrow was aspirated in a 1 mL syringe with a needle, and the contents of the bones were flushed into a Petri dish with 5 mL of medium. The medium was aspirated and flushed several times to disperse the bone marrow cells. The vertebrae were crushed with the backside of a 5 mL syringe in 5 mL of medium. The cell suspensions were filtrated with a 70 µm cell strainer (Falcon, USA). 5 mL of Lympholyte M (Cedarlane, Ontario, Canada) was pipetted into a 15 mL tube, and was overlayed carefully with 5 mL of cell suspension. After centrifugation for 20 min at 1,000 g, the cells at the interface of the Lympholyte M and medium were removed. The mononuclear cells were washed two times in 5 mL of medium. The cells were counted and the cell concentration was adjusted to 5×10^6^ /mL in complete culture medium consisting of high glucose DMEM supplemented with 15% FBS and 100 U/mL of penicillin-streptomycin and 2 mM L-glutamine, then cells were plated in plastic 10 cm plates. Non-adherent cells were removed after 6 h, the adherent cells were re-fed with complete DMEM medium, with additional media changes every 3-4 days. After approximately 8 days or when cell cultures reached confluence, cells were detached with 0.25% trypsin/0.02% EDTA solution (Biosharp, China) for 2 min and replated at 1×10^5^/mL, and were designated as passage 1 (P1) mMSCs. Passaging was performed every 3-4 days at a split ratio of 1:3. mMSCs were used at passages 3-5 for all experiments.
