# Supplementary material for: ER stress arm XBP1s plays a pivotal role in proteasome inhibition-induced bone formation
Source: Stem Cell Res Ther. 2020 Nov 30;11:516. doi: 10.1186/s13287-020-02037-3 (PMC7708206; doi:10.1186/s13287-020-02037-3)
Supplement: Supplementary file 7 — Additional file 7: Supplemental Table 1. List of antibodies used for Western blotting. [file 13287_2020_2037_MOESM7_ESM.docx]

**Supplemental Table 1. List of antibodies used for Western blotting.**

| **Primary Antibody** | **Dilution** | **Source** |
| --- | --- | --- |
| Mouse anti-beta actin (60008-1-Ig) | 1:2000 | Proteintech |
| Rabbit anti-BMP2 (18933-1-AP) | 1:1000 | Proteintech |
| Mouse anti-COL1A1 (sc-293182) | 1:500 | Santa Cruz Biotechnology |
| Mouse anti-OCN (sc-74495) | 1:1000 | Santa Cruz Biotechnology |
| Rabbit anti-OPN (22952-1-AP) | 1:1000 | Proteintech |
| Rabbit anti-RUNX2 (20700-1-AP) | 1:1000 | Proteintech |
| Rabbit anti-GRP78 (11587-1-AP) | 1:1000 | Proteintech |
| Rabbit anti-CHOP (15204-1-AP) | 1:1000 | Proteintech |
| Mouse anti-phospho IRE1α (NB100-2323) | 1:1000 | Novus Biologicals |
| Rabbit anti-IRE1α (NB100-2324) | 1:1000 | Novus Biologicals |
| Rabbit anti-XBP1s (25997-1-AP) | 1:1000 | Proteintech |
| Mouse anti-phospho PERK (649401) | 1:1000 | Biolegend |
| Mouse anti-phospho eIF2α (3398T) | 1:1000 | Cell Signaling |
| Mouse anti-ATF4 (sc-200) | 1:200 | Santa Cruz Biotechnology |
| Mouse anti-ATF6 (24169-1-AP) | 1:1000 | Proteintech |
| Goat anti-mouse-IgG-HRP (115-035-166) | 1:10000 | Jackson ImmunoResearch |
| Goat anti-rabbit-IgG-HRP (111-035-047) | 1:10000 | Jackson ImmunoResearch |
