# Supplementary material for: ER stress arm XBP1s plays a pivotal role in proteasome inhibition-induced bone formation
Source: Stem Cell Res Ther. 2020 Nov 30;11:516. doi: 10.1186/s13287-020-02037-3 (PMC7708206; doi:10.1186/s13287-020-02037-3)
Supplement: Supplementary file 8 — Additional file 8: Supplemental Table 2. Primer sequences used for Realtime PCR analysis. [file 13287_2020_2037_MOESM8_ESM.docx]

**Supplemental Table 2. Primer sequences used for Realtime PCR analysis.**

| **Gene symbol** | **Gen Bank Accession no.** | **Primer set sequence (5’->3’)** | **Amplicon size (bp)** |
| --- | --- | --- | --- |
| *Beta-actin* (m) | NM_007393 | Forward:  GCGACAGCAGTTGGTTGGAG  Reverse:  TTTGGGAGGGTGAGGGACTTC | 165 |
| *Bmp2* (m) | NM_007553.3 | Forward:  TTCCATCACGAAGAAGCC  Reverse:  GGACAGAACTTAAATTGAAGAAGA | 85 |
| *Col1a1*(m) | NM_007742.4 | Forward:  CTGGTGCGAAAGGTGAACC  Reverse:  AGCACCAACGTTACCCTGTA | 225 |
| *Osteocalcin* (m) | NM_007541 | Forward:  CAGTATGGCTTGAAGACC  Reverse:  CAGAGTTTGGCTTTAGGG | 80 |
| *Osteopontin* (m) | NM_001204201 | Forward:  AATGCTGTGTCCTCTGAA  Reverse:  GTCATCATCATCGTCATCAT | 108 |
| *Runx2* (m) | NM_001146038 | Forward:  GAACCAAGAAGGCACAGA  Reverse:  GGACACCTACTCTCTCATACTG | 109 |
| *Grp78* (m) | NM_001163434 | Forward:  GTCTGCTTCGTGTCTCCTCCTG  Reverse:  TCCTCCTTCTTGTCCTCCTCCTC | 220 |
| *Chop* (m) | NM_007837 | Forward:  TCAGTTATCTTGAGCCTAA  Reverse:  CTTCTGGAACACTCTCTC | 108 |
| *Atf4* (m) | NM_009716 | Forward:  TCTGCTGCTTACATTACTCTA  Reverse:  GATGCCACTGTCATTGTC | 75 |
| *Beta-actin* (h) | NM_001101 | Forward:  ATCGTGCGTGACATTAAGGAGAAG  Reverse:  AGGAAGGAAGGCTGGAAGAGTG | 179 |
| *GRP78* (h) | NM_005347 | Forward:  AGGAGGAGGACAAGAAGGAGGAC  Reverse:  CAGGAGTGAAGGCGACATAGGAC | 156 |
| *CHOP* (h) | NM_004083 | Forward:  TGCTTCTCTGGCTTGGCTGAC  Reverse:  CCGTTTCCTGGTTCTCCCTTGG | 145 |
| *ATF4* (h) | NM_001675 | Forward:  TCCGAATGGCTGGCTGTGG  Reverse:  AGTGTAGTCTGGCTTCCTATCTCC | 420 |
