# Supplementary material for: ER stress arm XBP1s plays a pivotal role in proteasome inhibition-induced bone formation
Source: Stem Cell Res Ther. 2020 Nov 30;11:516. doi: 10.1186/s13287-020-02037-3 (PMC7708206; doi:10.1186/s13287-020-02037-3)
Supplement: Supplementary file 9 — Additional file 9: Supplemental Table 3. Primer sequences used for ChIP assay. [file 13287_2020_2037_MOESM9_ESM.docx]

**Supplemental Table 3. Primer sequences used for ChIP assay.**

| **Primer**  **set no.** | **Primer set sequence (5’->3’)** | **Location** | **Amplicon size (bp)** |
| --- | --- | --- | --- |
| ChIP target region | Forward:  AGTGAGAGGCGGTTTGGTG  Reverse:  ACACCCGAGCACACAACTTA | Promoter:  -117 / +43 | 161 |
| ChIP nontarget region | Forward:  GAGCGCTAGGGTTGGGTT  Reverse:  AGAGGCTCTTTCCAACTCCA | Promoter:  -1218 / -1066 | 152 |
